# Concentration of trace elements in forest soil affected by former timber depot

**DOI:** 10.1007/s10661-020-08479-9

**Published:** 2020-09-14

**Authors:** Arkadiusz Warczyk, Tomasz Wanic, Jacek Antonkiewicz, Marcin Pietrzykowski

**Affiliations:** 1 Department of Ecology and Silviculture, Faculty of Forestry, University of Agriculture in Krakow, Al. 29 Listopada 46,, 31-425 Krakow, Poland; 2 Department of Agricultural and Environmental Chemistry, Faculty of Agriculture and Economics, University of Agriculture in Krakow, Al. Mickiewicza 21,, 31-120 Krakow, Poland

**Keywords:** Copper, Soil pollution, Timber depot, Monitoring

## Abstract

Certain parts of global forests show elevated concentrations of trace elements as a result of industry processes, places such as wood depots and plant protection products, which together degrade the forest environment. This paper examines a timber depot that used wood preservatives in World War II located in the Warcino Forest Inspectorate (Poland). It presents monitory findings on the degree to which the upper soil layer in the depot area has been contaminated by wood preservatives. Within two forest divisions, a network of soil extraction points was established, distinguished into three separate categories that demarcate the degrees of vegetation coverage and growth of the common pine. These were Area A (area with a pine stand that is several dozen years-old), Area B (areas with a pine stand that is approximately a dozen years-old) and Area C (areas without a pine stand). The Cu concentration in the surface categories was respectively 141.03/187.54/834.43 mg·kg^−1^. Above 600% in Cu concentration was noted in category C in comparison to category A. It was found that the content of most elements (B, Cd, Co, Cr, Fe, Mn, Mo, Ni, Pb, Zn) did not exceed the permissible values according to the Regulation of the Minister of the Environment on soil quality standards; however, in the case of Cu, the limit values established for forest and agricultural soils were exceeded, with the highest Cu contents found in the ‘C’ category. The results obtained confirm that the wood protection chemicals, such as copper sulphate, affected the long-term pollution of forest soils.

## Introduction

In recent years, environmental awareness has risen alongside a growing demand for wood products, inspiring a global aim to meet the demand for wood while minimising its environmental damage. The Food and Agriculture Organization (FAO) (2001) forecasts that demand for wood will increase by 45% to 2.3 billion cubic meters in 2020. Due to the heightened environmental awareness and the subsequent use of renewable raw materials to reduce greenhouse gas emissions and carbon sequestration from the atmosphere, timber has been of great interest to society for many years (Köhl et al. 2015; Ramage et al. 2017). While timber products are indeed made from renewable and sustainable environmental resources (Klein et al. 2016), their processing, like other products, nonetheless has different environmental impacts.

The processing of raw wood involves energy consumption and thus produces greenhouse gases (Le Quéré et al. 2009). Further environmental impacts of wood include the emissions involved in the transportation of timber products (Lindholm and Berg 2005), the use of chemicals and wood wastage (Jurgensen et al. 1997; Wootton 2012). Despite the availability of environmentally friendly wood preservatives, toxic agents such as chromated copper arsenate (CCA), cresote and preservatives comprised of volatile organic solvent (VOC)—all of which are washed out of the wood by numerous physical and chemical processes, leading to their entering, and thus contaminating, soil and groundwater—remain in use, although at restricted levels in Europe and the USA (Coggins 2008; Lin et al. 2009). Our current aim is to reduce the use of toxic wood preservatives through policies and legislation (Lin et al. 2009). At present, environmentally friendly wood preservatives are used to protect the wood, which reduce negative environmental impacts and improve the durability of wood products (Schultz and Nicholas 2002; Coggins 2008). The most common use of these preservatives is dry and includes copper-organic preservatives, which replace CCA, copper/chromium/boron (CCB) and copper/chromium/phosphorus (CCP) preservatives; organic fungicides and insecticides, which replace microemulsion water-dilutable concentrates and water and solvent-based coloured preservatives, which replace creosote (Coggins 2008).

While some scientific papers have addressed the impact of fungicides on various soil properties, microorganisms and human health (Pavlovic 2011, Liu et al. 2017, Li et al. 2019, Tang et al. 2019), the literature as a whole lacks information regarding the impact of chemical use in wood storage facilities on the surrounding soil environment.

Therefore, this research will focus on the environmental impacts of these facilities. In particular, it will focus on a former German wood warehouse used during World War II in the Warcino Forest District, where copper sulphate was used for wood preservation. Wanic et al. (2013) were the first to make the soil in the Warcino wood warehouse. According to many reference and report analysis, the study site is quite unique, mainly because the general level of forest soil Cu contamination was extreme.

The aim of this study is to assess the physicochemical properties and chemical contamination of forest soils due to preservation agents in the area of the historical wood storage site in the Warcino Forest Inspectorate.

## Materials and methods

### Area of study

**Fig. 1:**
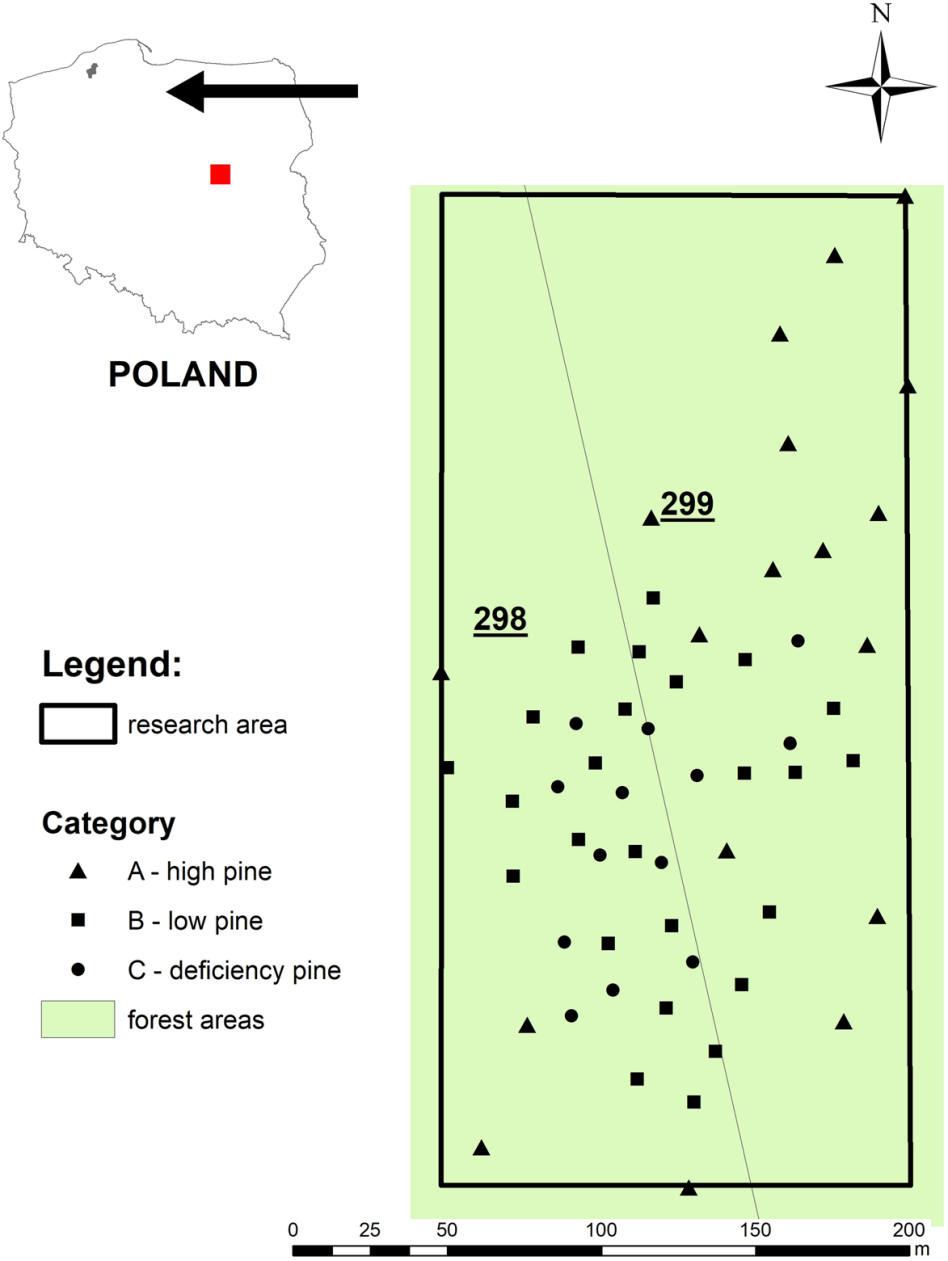
Fieldwork was carried out in 2017 in the Warcino Forest Inspectorate in the northern part of Poland, specifically within its divisions no. 299 and no. 298 [54°13′21.13″N 16°51′26.69″ E]

Fieldwork was carried out in 2017 in the Warcino Forest Inspectorate in the northern part of Poland, specifically within its divisions no. 299 and no. 298 [54°13′21.13″N 16°51′26.69″ E] (Fig. 1). During the World War II, there was a timber depot where CuSO4·5H2O wood preservatives were used.

To adjust to the identified morphology of the area and differences in herbaceous vegetation and stand coverage, an irregular grid of 57 sampling points was established (Fig. 1). Three plots demarcating different degrees of pine stands of varying health were distinguished, in which monitoring points were placed (Fig. 1):

- Area A: ‘Pine-high’—areas with a pine stand that is several dozen years old
- Area B: ‘Pine-low’—areas with a pine stand that is approximately a dozen years old, up to 6 m high, with features of weakness (atrophy of needles, decrease in the number of vintage years, small increments)
- Area C: ‘Pine-deficiency’—areas without any forest stand and with clearly inhibited plant growth

### Chemical analysis

In the lab, 57 soil samples from mineral soil horizons were dried and sifted through a 2-mm diameter sieve, while samples from organic soil horizons were ground up. To provide a brief overview of key metrics culled from chemical analyses of these samples and the methods used for their calculation:

- Particle size distribution was calculated using the aerometric method (fractions as in standard PN-R-04033)
- pH was calculated using the potentiometric method in H_2_O and 1 M KCl maintaining soil proportion: solution 1:2.5
- Organic carbon (C-org), total nitrogen (N-tot) and total sulphur (S-tot) content were calculated using a Leco CNS 2000 analyser (carbon and sulphur in the infrared, nitrogen in the thermal conductivity detector)
- Total alkalinity (TEB) was calculated by summing the exchangeable cations Ca2+, Mg2+, Na+ and K+ determined in an extract of 1 N CH_3_COONH_4_ with pH 7.0 using the AAS method. Exchangeable acidity (H_h_) of the soil was measured using 1 M Ca(OAc)_2_ extraction, followed by potentiometric titration to pH 8.2 with 0.1 m NaOH
- Cation exchange capacity (CEC) was defined as the equivalent sum of TEB and exchangeable acidity, and the base saturation (BS) as the sum of base cations as a percentage of CEC
- Soil samples were mineralised using microwave (MARS 5 apparatus), royal water was used as an extractor, and they were filtered into 50-ml flasks and the elements were determined with the ICP-OES Thermo 6000 series apparatus
- Spectrally pure reagents and Aldrich standard solutions were used in the chemical analyses

#### Quality control of the obtained measurement results

- Determinations of trace metals in each of the analysed samples were carried out in three replications. Accuracy of the analytical methods was verified by determining the samples of certified reference materials and standard solutions: CRM023–050-Trace Metals-Sandy Loam 7 (RT Corporation). The recovery within 80–120% of certified values was considered as acceptable.

### Statistical processing of results

The values were obtained using the Statistica line of analytical software (StatSoft Inc. 2013). Shapiro-Wilk test was employed to test normal distribution of the dataset, and homogeneity of variance was determined by the Levene’s test. Thereafter, one-way analysis of variance (ANOVA) was employed to compare the means of soil property differences among ecosystems. Tukey’s post hoc test at α ≤ 0.05 significance level was used to separate means. The RIR Tukey test was employed, preceded by a variance homogeneity test, to test the significance of the differences in mean copper values, *t*; the non-parametric Spearman’s rank test was used to analyse the trace elements.

## Results

### Physical and chemical properties of the soil

The analysed area was characterised by a dominant share of the following granulometric groups: clay sand and weak clay sand. Sandy clay was found where Cu accumulation was highest in the soil. Of 57 sampling points, the sand fraction (0.05–2.0) had the highest share, with the clay fraction lowest in the analysed level (1–7%). Three soil types were described on the analysed area: rusty soil (Area A), industrial soil (Area B) and urban soil (Area C). In accordance with the international classification World Reference Base for Soil Resources (WRB 2015), the marked soils were classified into the group Umbrisols and Technosols.

The analysed material was characterised by high variability of electrolytic conductivity, which ranged from 10.89 to 433.9 μS/1 cm (Table 1). The lowest value was recorded on the plot devoid of vegetation (Area C) and the highest value on the plot with limited pine stand growth (Area B).

**Table 1:** Physicochemical properties of the soil of the timber depot

Parameter	Unit	Mean	Minimum	Maximum	Standard deviation	Coefficient of variation (%)
EC (electrical conductivity)	μS/cm	53.46	10.89	433.90	88.76	166.03
pH KCl	–	4.05	3.50	5.76	0.34	8.33
pH H_2_O	–	5.08	4.22	6.56	0.43	8.39
Hh (exchangeable acidity)		4.86	3.02	9.31	1.49	30.70
TEB (total exchange bases)	mmol(+) ˑ kg-1	0.30	0.06	5.02	0.71	231.86
CEC (cation exchange capacity)		5.17	3.15	9.84	1.57	30.38
BS (base saturation)	%	5.23	1.28	61.18	9.05	172.96
C (carbon)	g/kg	11.5	5.4	26.5	4.6	39.63
N (nitrogen)	g/kg	0.6	0.3	11.4	0.02	37.73
C:N	–	19.76	14.74	25.17	2.62	13.28

**Fig. 2:**
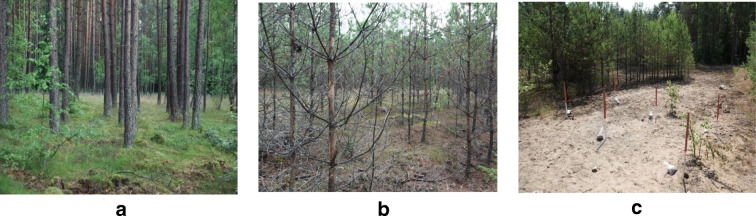
Area categories distinguished. Category A: Pine-high—areas with a pine stand that is several dozen years old; Category B: Pine-low—areas with a pine stand that is approximately a dozen years old, up to 6 m high, with features of weakness (atrophy of needles, decrease in the number of vintage years, small increments); Category C: Pine-deficiency—areas without any forest stand and with clearly inhibited plant growth

**Fig. 3:**
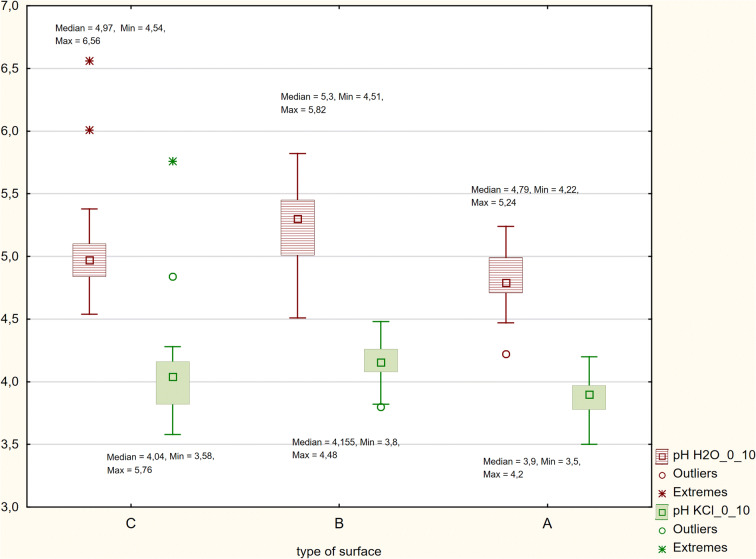
The soil pH ranged from strongly acidic (pH at KCl 3.65) to weakly acidic (pH at KCl 5.76)

The soil pH ranged from strongly acidic (pH at KCl 3.65) to weakly acidic (pH at KCl 5.76) (Figs. 2 and 3). Hh (exchangeable acidity) in the studied mineral level showed high variability in the field, with the maximum values in the healthy pine stand (Area A) reaching 9.31 mmol/kg^−1^ (Table 1). Lower acidity was recorded in Area B and Area C, where the reaction was only slightly acidic (Fig. 3).

The area under study showed little sorption capacity, with the content of total sorption capacity ranging from 3.15 to 9.84 mmol/kg^−1^ (Table 1). The highest *t*-value was recorded in the area with mature pine stands (Area A). In the case of the sum of alkaline exchange cations, the highest value was recorded at the site with the highest Cu concentration in the soil, and in the remaining area, it did not exceed 0.6 mmol/kg^−1^. The sum of alkaline cations reached the highest values in the area without vegetation (Area C).

The content of C-org in the analysed wood storehouse area was low, ranging from 5.4 to 26.5 g/kg for the whole area (Table 1). The highest C-org values were recorded in Area A and the lowest in Area C. The zone’s total N content ranged from 0.3 to 1.4 g/kg (Table 1). The lowest total N content was found within Area B, with the highest within Area A. The C/N ratio of the analysed upper soil levels was characterised by an average distribution of organic matter (C/*N* > 20), which corresponded to the majority of the studied zone. Only soils with a higher Cu content (Areas C and B) had a higher organic matter distribution (Table 1).

### Trace element content of topsoil layers

The elements analysed in the soil were characterised by high variability (Table 2), with the highest variability recorded for Cu and S. The content of B, Cd, Co, Cr, Fe, Mn, Mo, Ni, Pb and Zn was found to not exceed the limit values specified on soil quality standards in the Regulation of the Polish Minister of the Environment (Regulation 2016).

**Table 2:** Trace element content in soils

Parameters	Unit	Mean	Minimum	Maximum	Standard deviation	Coefficient of variation
Cu	mg kg^−1^	320.41 200*	2.74	4709.00	638.34	199.22
S		0.01	0.03	0.02	0.71	231.86
B		1.55 100*	0.74	2.90	0.70	44.89
Cd		0.12 2*	0.02	0.55	0.10	86.42
Co		1.75 50*	0.73	2.50	0.50	28.68
Cr		9.35 200*	4.55	31.38	4.77	51.01
Fe		5244.00	3954.00	6700.00	538.37	10.27
Mn		295.13	82.03	713.25	140.34	47.55
Mo		0.47 50*	0.24	1.74	0.31	66.02
Ni		4.41 150*	1.36	46.00	6.38	144.69
Pb		14.30 200*	8.36	67.13	8.25	57.68
Zn		12.92 250–300*	6.41	39.59	4.24	32.80

*Limitation values

The Cu content in the analysed surfaces of the historical timber depot fell within the range of 2.7–4709 mg kg^−1^ (Table 2). The permissible values of Cu in soil—200 mg·kg^−1^—were exceeded in all analysed research plots, especially in Areas C and B (Regulation 2016). Analysis of Cu content in Areas A, B and C showed a very high degree of variability (Table 3). The highest Cu content was found in Area C, where the content ranged from 146.43 to 4709.0 mg·kg^−1^, while the lowest content was recorded in Area A and ranged from 2.7 to 1041.8 mg kg^−1^.

**Table 3:** Cu concentration in the distinguished surface categories (mg kg^−1^)

Area	Number of sampling points	Mean	Minimum	Maximum	Standard deviation	Coefficient of variation
‘A’	18	141.03	2.74	1041.75	234.96	166.60
‘B’	26	187.54	53.93	723.00	139.28	74.27
‘C’	13	834.53	146.43	4709.00	1187.46	142.29

## Discussion

### Copper content in the tested areas

The analysed area showed reaction variations that ranged from strongly to weakly acidic, which in this geographical context affected the mobility and availability of metals for plants. This was particularly the case with the Cu content in the soil, which significantly exceeded the limit values specified in the Regulation of the Minister of the Environment (Regulation 2016). The concentration of Cu in the area of the wood component in the upper soil levels was exceeded in 43 out of 57 extraction points. Additional metrics used to assess the Cu content in the soil include the guidelines and threshold values proposed by Finnish and Swedish legislation on soil contamination (MEF 2007). The threshold value for Cu is 100 mg kg^−1^ and 150 mg kg^−1^ is considered as posing a risk to the environment and health (Adriano 2005). In this study, it was found that the average Cu contents in the studied Areas A, B and C also exceeded the threshold value of 100 mg kg^−1^ of soil. As indicated by Panagos et al. (2018), the average copper content in surface soil levels is below 10 mg kg^−1^ for Poland, Sweden and Denmark. The obtained results indicate that the Cu content in the studied areas was several times higher than the average Cu content found in Polish soils. Among the European countries, the highest Cu concentrations are found in the upper soil levels in Cyprus (53.41 mg kg^−1^). The average Cu content in soils of the European Union is 16.86 mg kg^−1^, while globally it is 30 mgˑkg^−1^ (Ballabio et al. 2018).

In the Spearman’s statistical test, there was a correlation between the content of Cd and Pb and Cu in Area C (Table 4). The correlation for Cd and Pb was 0.736 and 0.632, respectively (at the assumed level of significance α = 0.05). Significant differences were found between the types of surfaces due to the content of Cu in the upper soil levels. The highest content of this element was found in Surface C. Excessive concentration of this element hindered vegetation growth, which was visible in the field as parallel strips of land, was devoid of vegetation. Although the area is not used as a depot, there nevertheless exists a significant problem with Cu contamination of soils. The area of upper levels was composed mainly of granulometric groups of clay marker or weak clay sand. The average organic matter contents of Areas A and C were very similar, registering at 1.24 and 1.39 respectively. No significant dependence on grain size, pH and organic matter content in relation to Cu was found in the statistical analyses. This is probably because the highest concentration of Cu was found where the wood was stored (Area C). Approximate C-org content on the surface of Area A appeared to result from the presence of bark in the upper soil levels, which generally results in high Cu concentration, as decomposing organic compounds releases organic matter, which in turn increases the electron mobility of Cu. It is important to note that humic acids contained in peat soils may limit electron mobility or immobilise Cu in the soil profile (Fonseca et al. 2013). It is assumed that the use of copper sulphate as a wood preservative has contaminated the soil with copper.

**Table 4:** Correlation results using the non-parametric Spearma Rank test

Element	Area	Cu	S	B	Cd	Co	Cr	Mn	Mo	Ni	Pb	Zn
Cu	A		− 0.385	0.112	0.148	− 0.383	− 0.249	− 0.069	− 0.121	− 0.046	− 0.222	0.096
S	A	− 0.385		0.106	0.096	0.467	0.236	0.294	0.156	0.127	0.416	0.214
B	A	0.112	0.106		0.773	− 0.449	− 0.496	− 0.061	− 0.181	− 0.156	0.637	0.455
Cd	A	0.148	0.096	0.773		− 0.662	− 0.618	− 0.269	− 0.333	− 0.230	0.529	0.187
Co	A	− 0.383	0.467	− 0.449	− 0.662		0.738	0.626	0.505	0.156	− 0.088	0.209
Cr	A	− 0.249	0.236	− 0.496	− 0.618	0.738		0.333	0.858	0.414	− 0.160	0.044
Mn	A	− 0.069	0.294	− 0.061	− 0.269	0.626	0.333		0.228	0.139	− 0.110	0.589
Mo	A	− 0.121	0.156	− 0.181	− 0.333	0.505	0.858	0.228		0.418	0.088	0.119
Ni	A	− 0.046	0.127	− 0.156	− 0.230	0.156	0.414	0.139	0.418		− 0.154	− 0.183
Pb	A	− 0.222	0.416	0.637	0.529	− 0.088	− 0.160	− 0.110	0.088	− 0.154		0.245
Zn	A	0.096	0.214	0.455	0.187	0.209	0.044	0.589	0.119	− 0.183	0.245	
Cu	B		0.665	0.450	0.402	− 0.144	− 0.432	− 0.019	− 0.420	− 0.246	0.497	0.154
S	B	0.665		0.364	0.240	− 0.237	− 0.239	− 0.239	− 0.107	− 0.024	0.370	− 0.032
B	B	0.450	0.364		0.199	0.072	− 0.549	0.136	− 0.450	− 0.517	0.157	0.289
Cd	B	0.402	0.240	0.199		− 0.594	− 0.190	− 0.441	− 0.217	0.145	0.563	− 0.370
Co	B	− 0.144	− 0.237	0.072	− 0.594		0.431	0.785	0.337	− 0.121	− 0.457	0.586
Cr	B	− 0.432	− 0.239	− 0.549	− 0.190	0.431		0.110	0.892	0.612	− 0.164	− 0.126
Mn	B	− 0.019	− 0.239	0.136	− 0.441	0.785	0.110		0.075	− 0.375	− 0.430	0.681
Mo	B	− 0.420	− 0.107	− 0.450	− 0.217	0.337	0.892	0.075		0.568	− 0.181	− 0.118
Ni	B	− 0.246	− 0.024	− 0.517	0.145	− 0.121	0.612	− 0.375	0.568		0.023	− 0.648
Pb	B	0.497	0.370	0.157	0.563	− 0.457	− 0.164	− 0.430	− 0.181	0.023		− 0.212
Zn	B	0.154	− 0.032	0.289	− 0.370	0.586	− 0.126	0.681	− 0.118	− 0.648	− 0.212	
Cu	C		0.626	0.527	0.736	− 0.033	0.264	− 0.231	− 0.330	− 0.099	0.632	0.440
S	C	0.626		0.110	0.286	0.159	0.308	0.148	0.033	0.423	0.478	0.341
B	C	0.527	0.110		0.764	− 0.648	− 0.324	− 0.648	− 0.648	− 0.731	0.225	0.115
Cd	C	0.736	0.286	0.764		− 0.368	− 0.132	− 0.247	− 0.549	− 0.429	0.434	0.242
Co	C	− 0.033	0.159	− 0.648	− 0.368		0.885	0.665	0.731	0.742	0.396	0.654
Cr	C	0.264	0.308	− 0.324	− 0.132	0.885		0.396	0.659	0.549	0.681	0.830
Mn	C	− 0.231	0.148	− 0.648	− 0.247	0.665	0.396		0.692	0.753	− 0.104	0.231
Mo	C	− 0.330	0.033	− 0.648	− 0.549	0.731	0.659	0.692		0.632	0.093	0.308
Ni	C	− 0.099	0.423	− 0.731	− 0.429	0.742	0.549	0.753	0.632		0.066	0.280
Pb	C	0.632	0.478	0.225	0.434	0.396	0.681	− 0.104	0.093	0.066		0.758
Zn	C	0.440	0.341	0.115	0.242	0.654	0.830	0.231	0.308	0.280	0.758	

### The influence of applied wood protection agents on the chemical properties of soils

The varying levels of the elements in the surfaces of Areas A, B and C of the former timber depot can be partly explained by the presence of human activity in this area. The concentration of boron in the analysed area was minimal, amounting to 0.74–2.9 mgˑkg^−1^, compared to an average concentration in Polish soils of 10–100 mg kg^−1^ (Kabata-Pendias 2010). The content of Co in soils depends on the type of parent rock, as well as on the presence of iron and manganese compounds. The Forum of European Geological Surveys (FOREGS; Salminen et al. 2005) determined the average Co content of European soils to be 1–20 mg kg^−1^; Alloway (2013) conversely has determined that the average content is 7 mg kg^−1^ in Europe and 1.1 mg kg^−1^ worldwide. The Finnish Regulation of the Minister of the Environment of the MEF (2007) recognises that the concentration of Co in soil above 100 mg kg^−1^ threatens the environment. In the soil analysed, the concentration of Co reaches 0.73–2.5 mg kg^−1^, and therefore, the soil in the area surrounding the wood depot has acceptable levels of this element (2–100 mg kg^−1^) (Adriano 2005).

The occurrence of Mn in soils depends on the content in the parent rock, as well as on soil-forming processes that determine the profile distribution of this element. The average content for different types of European soils is 373.05 mg kg^−1^ (Tóth et al. 2016), whereas globally, it is 418 mg kg^−1^ (Alloway 2013). The concentration of Mo on the depot was 0.24–1.74 mg kg^−1^; on sandy soils, it is usually 0.1–3.7 mg kg^−1^ (Kabata-Pendias 2010). The surfaces analysed contained Mn and Mo in the amounts reported by the above-mentioned authors.

The average Ni content of soils in Europe is 18 mg kg^−1^ (Tóth et al. 2016), while according to MEF (2007), it is 3–100 mg kg^−1^, and in the world, it is approximately 18 mg kg^−1^ (Alloway 2013). Ni concentrations in soils above 100 mg kg^−1^ pose a risk to the environment (MEF 2007). Ni content in the analysed Areas A, B and C was within the range given for Polish soils (Regulation of the Minister of Environment 2016).

Zn is very common in the environment, but its differentiation depends on geological and anthropogenic conditions. Natural contents of Zn in soils are 8–110 mg kg^−1^ (MEF 2007), 48 mg kg^−1^ (Alloway 2013) and 62 mg kg^−1^ for soils globally (Alloway 2013). Zn content in Polish soils is 50–100 mg kg^−1^. Zn concentration (6.41–39.59 mg kg^−1^) on the analysed wood component was within the accepted ranges (Kabata-Pendias 2010). The Zn content in the soil depends on the pH and granulometric properties of the soil: the looser the soil and the more acidic the soil reaction, the lower the values. Poor soils are deficient in this element (Alloway 2004).

The natural concentrations of Cd, Cr and Pb in soil are respectively 0.01–0.15 mg kg^−1^, 6–170 mg kg^−1^and 0.1–5 mg kg^−1^. Exceeding 10 mg kg^−1^, 200 mg kg^−1^ and 750 mg kg^−1^, respectively, threatens the environment (MEF 2007). The permissible contents of Cd, Cr and Pb, according to the Regulation of the Minister of the Environment of 2016, were not exceeded.

The activity of the former wood depot and the use of chemicals for wood preservation significantly affected the phytotoxic Cu contamination of the soil as a result of the use of copper sulphate. Despite the passage of more than 70 years, the concentration of Cu in the soil solution is still high. This is related to the low pH of the soil, the content of organic matter in the soil and the influence of grain size distribution. In this case, it is advisable to apply remediation to restore the soil’s utilisation value and to monitor the contaminated area (Park et al. 2011; Bolan et al. 2014; Yuan et al. 2015; Caporale and Violante 2016; Cristaldi et al. 2017; Liu et al. 2018; tremblay et al. 2019).

## Conclusions

The content of Cu in the designated plots differed significantly; Area C had the highest concentration of copper, while Area A had the lowest concentration. In the majority of the analysed samples, Cu content exceeded 200 mg kg^−1^. The results obtained confirm the permanent chemical degradation of the soil environment caused by the applied wood preservatives. To restore the productive value of the soil, it is recommended that phytoremediation is carried out by planting trees.
